# The relationship between parent mental health and intimate partner violence on adolescent behavior, stigma and school attendance in families in rural Democratic Republic of Congo

**DOI:** 10.1017/gmh.2018.10

**Published:** 2018-05-21

**Authors:** Nancy Glass, Anjalee Kohli, Pamela J. Surkan, Mitma Mpanano Remy, Nancy Perrin

**Affiliations:** 1 Johns Hopkins University School of Nursing, Baltimore, Maryland, USA; 2 Center for Health Research, Kaiser Permanente Northwest, Portland, Oregon, USA; 3 Johns Hopkins Bloomberg School of Public Health, Bukavu, Democratic Republic of Congo; 4 Programme d'Appui aux Initiatives Economiques (PAIDEK), Bukavu, Democratic Republic of Congo

**Keywords:** Intimate partner violence, mental health, post-conflict, stigma, young adolescents, AYPA, African Youth Psychosocial Assessment, CPT, cognitive processing therapy, DRC, Democratic Republic of Congo, HDDS, Household Dietary Diversity Scale, HSCL, Hopkins Symptom Checklist, HTQ, Harvard Trauma Questionnaire, IPV, intimate partner violence, IRB, Internal Review Board, NICHD, National Institute of Child Health and Human Development, NIH, National Institute of Health, PFP, pigs for peace, PTSD, post-traumatic stress disorder, RA, research assistant, RFR, rabbits for resilience

## Abstract

**Background.:**

Prolonged conflict and economic instability challenge the existing support networks in families and society places significant stress on both adults and adolescents. Exploring individual, family and social factors that increase the likelihood of or protect adolescents from negative outcomes are important to the development of evidence-based prevention and response programing in global settings.

**Objective.:**

Examine the relationship between parent mental health and experience/perpetration of intimate partner violence (IPV) and adolescent behaviors, stigma, and school attendance. The relationship is further examined for differences by gender.

**Methods.:**

Secondary analysis of data from an ongoing comparative effectiveness trial of a productive asset transfer program in eastern Democratic Republic of Congo (DRC).

**Results.:**

Three hundred and eighty-eight adolescent and parent dyads were included in the analysis. The analysis demonstrated that parent mental health and IPV can have a negative impact their children's well-being and the impact is different for boys and girls, likely linked to gender roles and responsibilities in the home and community. Social relationships of adolescents, as reported through experienced stigma, were negatively impacted for both boys and girls. Parent report of symptoms of PTSD and depression had a stronger negative effect on girls’ outcomes, including experienced stigma, externalizing behaviors, and missed days of school than boys. For adolescent boys, their parent's report of IPV victimization/perpetration was associated with more negative behaviors at the 8-month follow-up assessment.

**Conclusion.:**

The findings reinforce the critical importance of interventions that engage parents and their children in activities that advance health and improve relationships within the family.

## Introduction

Although adolescents (defined as youth between 10 and 19 years of age) represent about one-fourth of the world's population (Sawyer *et al.*, 2012) with almost 90% living in LMICs (low- and middle-income countries), most research focused on adolescence takes place in high-income countries (Fulu *et al.* 2017). Even less visible in the research discourse and policy foci are young adolescents aged 10–15 years (Chong *et al.* 2006) and those living in conflict-affected settings (Karibu *et al.* 2013; Okello *et al.* 2014). Elevated distress and unstable social and economic conditions are common in conflict and post-conflict settings. Prolonged conflict and economic instability challenge the existing support networks in families and society and can place significant stress on both adults and adolescents. Adolescence is a life stage marked by significant biological, social, emotional, and psychological changes. The young adolescent development period (youth age 10–15 years) is an important period and yet often overlooked by interventions and research (Bonomo *et al.* 2004; Sommer, 2011). There is a developing consensus among practitioners and researchers that armed conflict directly and indirectly, through the negative effects on family, social and economic conditions, impacts adolescent health and well-being (Miller & Jordans, 2016). This consensus expands the previous focus of predictors of adolescent mental health and well-being in conflict and post-conflict settings to include, for example, parent and peer relationships and economic opportunities (Miller & Rasmussen, 2010; Miller & Jordans, 2016). Although most youth demonstrate resilience in the face of adversity (Luthar *et al.* 2000; Betancourt & Khan, 2008; Karibu *et al.* 2013), exploring individual, family, and social factors that increase the likelihood of or protect adolescents from mental distress is important (Tol & van Ommeren, 2012). Such information would contribute to the development of evidence-based promotion and prevention programming in diverse global and humanitarian settings.

The ecological model of human development proposes that multiple and multi-level factors at the individual, microsystem (e.g. family and peer relationships), mesosystem and macro level influence child development outcomes (Bronfenbrenner & Morris, 2007). These levels overlap, influencing each other to yield outcomes and highlight opportunity for prevention. This paper examines two levels of the ecological model of child development: the individual and the relationship levels, specifically the parent–child relationship.

Research primarily conducted in high-resource settings provides evidence for the importance of parental attachment for positive child and adolescent development (Fulu *et al.* 2017). For example, in non-conflict and conflict settings, being depressed as a parent can negatively impact parenting, family functioning, parent–child relationships and have consequences on child development, behavior and cognitive function (Wachs *et al.* 2009; Masten & Narayan, 2012). Among adults, often parents, living in conflict or post-conflict areas, experiences of trauma and displacement are common and are associated with elevated symptoms of depression and post-traumatic stress disorder (PTSD) (Miller *et al.* 2008; Roberts & Browne, 2011). In eastern Democratic Republic of Congo (DRC), rural populations in South Kivu Province, the location of the present study, have experienced more than two decades of conflict, instability, and extreme poverty (Wakabi, 2008; United Nations Development Program, 2015; Glass, 2017). Estimates of exposure to traumatic events and human rights violations among adults, adolescents, and children are high with most reporting exposure to multiple different traumatic events, including torture, deprivation (e.g. absences of food, housing, water, and medical care), gender-based violence, including sexual exploitation and violence (Johnson *et al.* 2010; Glass *et al.* 2014; Glass, 2017). In 2010, a population-based cross-sectional survey in conflict-affected areas of eastern DRC reported a high prevalence of male and female adults meeting the criteria for major depression (41%) and PTSD (50%) (Johnson *et al.* 2010). Further, there is an increased risk for intimate partner violence (IPV) post-conflict related to experiences of trauma and stress (e.g. male and female experiences of deprivation, violence, and displacement, increased alcohol consumption to cope with stress and loss, lack employment and income generating activities resulting in financial distress) associated with loss of material resources, social status, and well-being (Ezard *et al.* 2011). In the DRC, 68.2% of women between ages 15 and 49 years report the experience of physical, sexual, or emotional intimate partner violence (Tlapek, 2014). Women who experience IPV may report symptoms consistent with depression, anxiety and PTSD (Ellsberg *et al.* 2008) and given the symptoms (e.g. loss of concentration, lack of sleep, loss of appetite, and hypervigilance) associated with these mental health issues, mothers may experience reduced parenting capacity and resources to care for children. Consequently, adolescents may feel less trust and security in their own family. Youth growing up in homes where a parent experiences or perpetrates IPV are at risk for abuse by the parent and for developing emotional and behavioral problems (e.g. internalizing and externalizing behaviors) during their childhood. Evidence has consistently demonstrated that witnessing IPV in childhood increases the risk of victimization or perpetration of IPV and other forms of violence in adult relationships (Holt *et al.* 2008; Fulu E *et al.* 2013; Fulu *et al.* 2017). For example, witnessing IPV in the home may result in adolescents demonstrating aggressive behaviors, isolating themselves from friends and others to hide experiences of family violence, experiencing difficulty in maintaining peer relationships, using avoidant attachment styles in their own relationships and engaging in harmful behaviors such as unsafe sex, drug, and alcohol use (Kitzmann *et al.* 2003; Wolfe *et al.* 2003; Holt *et al.* 2008; Fulu *et al.* 2017). In a qualitative study with adult men and women reporting perpetration or experience of IPV in eastern DRC, participants expressed concern about the impact of IPV on adolescents’ health and their future relationships and opportunities. Both men and women in abusive relationships explained that IPV affected the quality of their parenting, their children's feelings of security in the home and led to stigma by other family members, neighbors, and community members. Parents expressed concern on how witnessing IPV may place children at risk for behavioral issues such as alcohol consumption and use of violent behaviors at home and in the community (Kohli *et al.* 2015). Studies with adolescents including those in conflict and post-conflict settings indicate the importance of family and community connectedness (Betancourt *et al.* 2012) and social support to adolescent mental health, including externalizing and/or internalizing behaviors. Adolescent's use of internalizing and externalizing behaviors is often an expression of disorder or insecurity in the home and the behaviors differ by gender given the socially defined roles and responsibilities of girls and boys (Llabre & Hadi, 1997; Kliewer *et al.* 2001). In addition, parents participating in our livestock productive asset transfer program requested programming to engage young adolescents in activities to improve their educational opportunities and encourage healthy behaviors.

Therefore, the purpose of the paper is to examine the: (1) relationship of parent mental health and IPV on adolescent experienced stigma, externalizing behaviors and past month school attendance at 8-month post-baseline; and (2) relationship between parent mental health and IPV on adolescent behavior, stigma, and school attendance by gender.

## Methods

### Study design

This is a secondary data analysis of a National Institute of Health (NIH)/National Institute of Child Health and Human Development (NICHD)-funded trial (NCT02008695). The study evaluates the effectiveness of productive asset transfer program interventions that engage both adolescents [rabbits for resilience (RFR)] and their parents [pigs for peace (PFP)] on outcomes of economic stability, health, and gender equality (as measured by reduction in IPV, girls education) compared with adolescent only (RFR) intervention or adult only (PFP) intervention only. The study protocol has been described in detail elsewhere (Kohli *et al.* 2017). In this paper, we examine the parents’ report of symptoms of PTSD and depression and IPV at baseline with their adolescent children's report of behavior, stigma and days missed from school at 8-month follow-up. This lagged measurement approach reduces some of the common method variance associated with cross-sectional analyses. The timing of follow-up data collection periods is based on key intervention time points (e.g. the birth of livestock/animal offspring, reimbursement of livestock/animal loan) (Kohli *et al.* 2017). At 8-month follow-up, the PFP intervention with parents had not been fully implemented.

### Setting and sample

The PFP livestock productive asset transfer program is being conducted in ten villages of the Walungu and Kabare Territories in the South Kivu Province of eastern DRC. The villages were selected for participation in the program based on (a) feasibility of delivering an intervention over a wide geographic area; (b) village leadership commitment to the intervention and research; and (c) existing relationships by the Congolese research team with village leadership and services. Male and female adolescents, between 10 and 15 years of age were eligible for participation in the adolescent productive asset transfer program (RFR) only if their parents had been randomized to and enrolled in the delayed control groups of the PFP program. To be eligible, male or female children expressed an interest and commitment to the productive asset transfer program [e.g. participation in building rabbit hutch, attend training and group meetings related to caring for rabbits and repayment of rabbit offspring and transfer of asset (rabbit) to other adolescents in family and community] and were living in the household with the parent. Parents in the PFP program provided consent for their eligible child prior to the children being approached for assent. A total of 509 adolescents assented and completed the baseline survey and 94.1% (*n* = 479) parents’ consented and completed the adult survey at baseline. Of the 479 youth whose parent completed a baseline survey, 380 (79.3%) completed the 8-month follow-up survey that was used for this analysis.

### Intervention overview

As the intervention has been detailed elsewhere, we will provide only a brief overview (Glass, 2017; Kohli *et al.* 2017). The two productive asset transfer programs are PFP and RFR and were developed over a 10-year collaboration and partnership between Johns Hopkins University School of Nursing and Programme d'Appui aux Initiatives Economiques du Kivu (PAIDEK), a leading microfinance organization in eastern DRC (Glass *et al.* 2012). RFR, a youth-led productive asset program involves male and female youth between 10 and 15 years of age with permission of a parent. Youth in the program receive a productive asset loan of a female rabbit after participating in an educational program to learn about caring for and breeding healthy rabbits as well as committing to building a rabbit hutch. With the help of the RFR program mentors, the rabbits are breed and when the rabbit gives birth the first time, youth reimburse the program with two female rabbit offspring, which are then transferred to other youth (ages 10–15 years) in the same family or same community. Once their two rabbits are repaid, youth retain the original rabbit and the remaining offspring to continue breeding with the support of RFR staff, as well as sell at the local market for approximately US$10.

PFP is an adult-led asset transfer program and uses a similar approach to economic development as RFR; however, in PFP, the productive asset transfer is a piglet between 2 and 4 months old and is provided to a male or female adult head of household. Adult participants receive the productive asset loan after the pigpen is built and they completed training on how to raise, care for and breed pigs. Once the pig breeds and gives birth, the loan recipient repays the loan by transferring to two piglet offspring to other families in the same village (Glass *et al.* 2014).

### Data collection

Data collected occurred at baseline (e.g. after PFP/RFR group formation but prior to initiation of intervention) and 8-month post-baseline. Questionnaires were developed after reviewing existing, validated research instruments used in similar settings, discussion with the Congolese partners and findings from this team's previous research (Glass *et al.* 2014; Glass, 2017). Translation and back translation of the questionnaire from English to French was completed and then translated to Mashi and Swahili, local languages in project areas. Ten male and female research assistants (RAs) were trained by our five Congolese supervisors to conduct interviews using the study questionnaire; training included a focus on research ethics and discussion of sensitive topics (e.g. trauma, intimate partner violence, referral to services for participants as appropriate). Trained RAs pilot tested the consent/assent and a questionnaire on a tablet computer with female and male adults and adolescents living in villages outside the study setting. The pilot testing focused on appropriateness and comprehension of the questionnaire and study protocols (Glass, 2017).

### Ethics

The Johns Hopkins Medical Institute Internal Review Board (IRB) approved this protocol. Due to a lack of a local Congolese ethics review board, a committee of respected Congolese educators at Université Catholique de Bukavu and community members reviewed the research and intervention protocols before giving approval for this study. Pilot and study interviews were conducted after taking voluntary, informed, oral consent from the parent. For youth, interviews were initiated after taking voluntary, informed, oral consent from parents for their child, and assent from the adolescent participant. Interviews were conducted during times when adults would be earning daily income and youth would be contributing to household activities (e.g. getting water, doing household chores, and herding animals). To compensate for time (45–90 min) spent away from work and household activity, participants that completed the interview received approximately 1.50 USD, an amount consistent with incentives for other studies in the area (Glass, 2017).

### Study questionnaire

The parent and adolescent questionnaire adapted validated measures that have been used in studies in similar settings (García-Moreno *et al.* 2005; Pronyk *et al.* 2006).

### Food security

The Household Dietary Diversity Scale (HDDS)(Swindale & Bilinsky, 2006) assessed the total number of food groups (range: 0–12 items) consumed by household members in the previous day and night and was completed by parents. Food security as measured by the HDDS is ‘used as a proxy measure of the socio-economic level of the household’ (Swindale & Bilinsky, 2006).

### Parent mental health

Parent PTSD and depression were measured using validated instruments that have been widely used in sub-Saharan Africa and in conflict settings, including DRC (Mollica *et al.* 1993; Mollica *et al.* 2004; Roberts *et al.* 2008). A 16-item version of section four of the Harvard Trauma Questionnaire (HTQ) assessed the frequency of symptoms consistent with PTSD in the past 7 days. A 15-item depression component of the Hopkins Symptom Checklist (HSCL) was used to understand participant experience of symptoms that distressed or bothered them in the past four weeks. In both HSCL and HTQ, participants rate the frequency of experiencing each symptom from 1 (not at all) to 4 (a lot). Both the HTQ and HSCL show strong psychometric properties in conflict and humanitarian settings (Sabin *et al.* 2003; Ventevogel *et al.* 2007; Roberts *et al.* 2008). In this sample, Cronbach's alpha was 0 0.85 for depression and 0.89 for PTSD.

### Parent experience/perpetration of IPV

An adapted version of the conflict tactics scale was used to measure physical, psychological, and sexual intimate partner violence. The conflict tactics scale is a widely used measure of IPV and includes questions on specific acts of violence (Straus, 1990). At baseline, female partners were asked about their experience of specific acts of controlling behavior (six items), psychological abuse (two questions), physical violence (eight items) and sexual violence (two items) perpetrated by their male partners in the past 12 months (World Health Organization, 2013). Male parents were asked the same questions about their perpetration of controlling behaviors, psychological, physical, and sexual violence against their female partners in the past 12 months. For this analysis, the count of the number of items experienced or perpetrated within each category of controlling behavior, physical, sexual, or psychological IPV in the past 12 months were used.

### Adolescent lifetime traumatic events

An 18-item version of the HTQ (Mollica *et al.* 2004) was used during baseline interview to assess youth lifetime experience of traumatic events.

### Adolescent experienced stigma

We adapted a measure of everyday discrimination (Essed, 1991; Williams *et al.* 1997) to look at ‘chronic, routine, and relatively minor experiences of unfair discrimination’ (Williams *et al.* 1997) (i.e. experienced stigma) by adolescent participants. Youth answered eight questions (e.g. people act as if they are afraid of you, people treat you with less respect than others, you are called names or insulted) on a three-point scale (e.g. never, sometimes, and always) about the frequency of different types of experienced stigma occurring in their day-to-day life. Cronbach's alpha in this sample was 0.79.

### Externalizing behaviors

The African Youth Psychosocial Assessment (AYPA) (Betancourt *et al.* 2014) was adapted for use in the DRC. The AYPA measures experience or feelings in the past 7 days on a four-point scale: never, sometimes, often, and always. This analysis uses the externalizing behaviors subscale. Ten questions were used to assess externalizing behaviors (e.g. fighting, using bad language, being disrespectful, Cronbach's alpha = 0.79). An average score was calculated and used for this analysis with increasing values representing more externalizing behaviors.

### Days missed from school

Youth enrolled in school answered questions about the number of days of school that they missed in the past 1-month measured on a 1–4 point scale (i.e. 0 days, 1–2 days, 3–5 days, and 6 or more days missed from school). The 8.1% of adolescent participants that were not enrolled in school were assigned to the 6 or more days missed from school in the past 1-month category.

### Statistical analyses

All analyses control for adolescent age, food security as a proxy for socio-economic status, number of lifetime traumatic events the adolescent had experienced, and intervention group assignment in the parent study (dummy coded). To examine the effect of parent baseline PTSD and depression on adolescent outcomes (experienced stigma, externalizing behaviors) 8-month post-baseline, multiple linear regression was used. Ordinal regression was used to explore the relationship between parent baseline PTSD and depression on the adolescent outcome 8-month post-baseline of days missed from school. Due to the correlation between PTSD and depression, analyses of the relationship between parent PTSD and depression with adolescent outcomes were tested in separate models. A Bonferroni-adjusted alpha level for these analyses of 0.017 was used for this set of three outcomes (i.e. externalizing behavior, experienced stigma, days missed from school). Hierarchical multiple linear and ordinal regression were used to examine the effect of parent IPV on these same three adolescent outcomes in the subset of the sample where the parents were married/partnered. Covariates of child age, food security, number of traumatic events experienced by the youth, and intervention group was entered in the first step and the IPV count variables were entered in the second step. SPSS version 24 (IBM Corp, 2016) was used for all statistical analyses.

## Results

Of the 388 adolescents (*N* = 192 boys, *N* = 188 girls), more than half of male and female adolescents were between 10 and 12 years of age at baseline (53.7% and 52.1%, respectively) (Table 1). Adolescents had experienced an average of two traumatic events in their lifetime (2.37 for boys; 2.47 for girls). At baseline, approximately one-third of adolescents reported that they had missed no school days in the past month (31.9% boys; 33.5% girls). Experiences of stigma (e.g., people act as if they are afraid of you, people treat you with less respect than others, you are called names or insulted) were similar for male (*M*=1.28 at baseline, M=1.17 at 8-month follow up) and female (*M*=1.27 at baseline, *M*=1.13 at 8-month follow up) (Table 2). Boys had slightly higher scores than girls (1.27 *v.* 1.23, respectively) on externalizing behaviors (e.g. fighting, using bad language, being disrespectful). Slightly more than half (53.9%) of adolescents reported the primary caregiver as a parent, the remaining participant's guardians was another adult family member except for 1.1% (*n* = 4 adolescents) that reported other community members to be their guardian.

**Table 1.** Adolescent and parent/guardian demographics and health

Adolescent characteristics		
Baseline	Boys (*N* = 192)	Girls (*N* = 188)
Age group		
10–12 years	103 (53.7%)	98 (52.1%)
13–15 years	89 (46.3%)	90 (47.9%)
Average number of lifetime traumatic experiences (standard deviation)^a^	2.37 (2.03)	2.47 (2.02)
Household dietary diversity score (standard deviation)^a^	3.26 (1.56)	3.45 (1.88)
Days missed from school^b^		
0	61 (31.9%)	63 (33.5%)
1–2	52 (27.2%)	58 (30.9%)
3–5	43 (22.5%)	39 (20.7%)
6 or more	35 (18.3%)	28 (14.9%)
Experienced stigma^a^	1.28 (0.35)	1.27 (0.32)
Externalizing behaviors^a^	1.27 (0.30)	1.23 (0.23)
Parent/guardian characteristics	Men (*N* = 44)	Woman (*N* = 336)
Percent of parents/guardians that are female	85.4%	88.4%
Age Group		
15–19 years	0	2 (0.6%)
20–24 years	1 (2.3%)	25 (7.4%)
25–34 years	3 (6.8%)	97 (28.9%)
35–44 years	9 (20.5%)	104 (31.0%)
45–60 years	24 (54.5%)	97 (28.9%)
61 + years	7 (15.9%)	11 (3.3%)
Marital status		
Married	43 (97.7%)	234 (69.6%)
Widowed	1 (2.3%)	72 (21.4%)
Divorced/separated	0	19 (5.7%)
Abandoned	0	8 (2.4%)
Never married	0	3 (0.9%)
Average past month depression symptom score (standard deviation)^a^	1.55 (0.48)	1.70 (0.47)
Average past week post-traumatic stress symptom score (standard deviation)^a^	1.69 (0.41)	1.90 (0.49)
IPV Mean number of items (standard deviation)		
Controlling behavior^a^	0.93 (0.99)	1.61 (1.24)
Psychological abuse^a^	0.40 (0.69)	0.46 (0.69)
Physical abuse^a^	0.14 (0.41)	0.29 (0.86)
Sexual abuse^a^	0.09 (0.36)	0.18 (0.46)

a Range of possible responses: youth lifetime traumatic experiences between 0 and 18 events; Household dietary diversity score between 0 and 12 different food items; Average externalizing behavior score between 1 and 4; Experienced stigma score between 1 and 3; Parent/guardian average past month depression symptom score (HSCL) between 1 and 4; Parent/guardian past week post-traumatic stress symptoms score (HTQ) between 1 and 4; Controlling behaviors 0–6; Psychological abuse 0–2; Physical abuse 0–8; Sexual abuse 0–2. For all measures, increasing number means increasing events/items/symptoms. ^b^Days missing from school are missing on 1 boy.

Of the 388 parents that completed baseline and 8-month follow-up questionnaires, 336 were female and 44 male. Median age was between 45 and 60 years old for men and 35 and 44 years old for women. The vast majority (97.7% of men and 69.6% of women) was married at baseline. Women reported more symptoms consistent with depression than (1.70 *v.* 1.55) and PTSD (1.90 *v.* 1.69) than men. Among the 270 married/partnered parents at baseline, women reported experiencing more IPV than men reported perpetrating. Controlling behavior was the most common form of IPV reported with 82.4% of women reporting experiencing controlling behaviors and 58.1% of men reporting perpetrating at least one type of controlling behavior, followed by psychological abuse (36.1% women experience, 27.9% men perpetrate), sexual abuse (14.5% women experience, 7.0% men perpetrate) and physical abuse (14.1% women experience, 2.1% men perpetrate). Table 1 presents the mean number of items experienced/perpetrated in each category.

### Relationship of parent mental health on adolescent experienced stigma, behavior, and days missed from school

For boys, parents/guardian with symptoms consistent with PTSD at baseline were significantly more likely to report experienced stigma (e.g. people act as if they are afraid of you, people treat you with less respect than others, you are called names or insulted) at 8-month post-baseline (*B* = 0.214, *p* = 0.003). However, parent symptoms consistent with PTSD were not associated with boys’ report of externalizing behavior or days missed from school. Parent depression was not related to experienced stigma, externalizing behavior, or days missed from school at 8-month follow-up for boys (Table 3).

**Table 2.** Adolescent behavior at 8- month post-baseline

	Boys (*N* = 192)	Girls (*N* = 188)
Days missed from school^b^		
0	64 (37.9%)	68 (42.0%)
1–2	48 (28.4%)	49 (30.2%)
3–5	43 (25.4%)	26 (16.0%)
6 or more	34 (18.0%)	40 (21.9%)
Experienced stigma^a^	1.17 (0.27)	1.13 (0.28)
Externalizing behaviors	1.19 (0.21)	1.20 (0.22)

a Range of possible responses: Average externalizing behavior score between 1 and 4; Experienced stigma score between 1 and 3. For all measures, increasing number means increasing days missed/behaviors/stigma; ^b^Days missed from school was missing on three boys and five girls.

**Table 3.** Parent/guardian mental health at baseline relationship with child behavior at 8 months

	Experienced stigma^a^		Externalizing behavior		Days missed from school	
	B	*p* value	B	*p* value	B	*p* value
Boys (*N* = 191)						
Parent/guardian PTSD	**0**.**214**	**0**.**003**	0.048	0.515	0.459	0.111
Parent/guardian depression	0.124	0.088	−0.075	0.310	0.063	0.835
Girls (*N* = 186)						
Parent/guardian PTSD	0.116	0.119	**0.209**	**0.005**	**0.752**	**0.008**
Parent/guardian depression	**0.188**	**0.011**	**0.174**	**0.019**	0.476	0.083

a All analyses control for intervention group, youth trauma experiences, food security, and youth age.

**Table 4.** Parent/guardian experience/perpetration of IPV at baseline relationship with child behavior at 8 months

	Experienced stigma^a^		Externalizing behavior		Days missed from school	
	B	*p* value	B	*p* value	B	*p* value
Boys (*N* = 132)						
Controlling behavior^a^	**0**.**278**	**0**.**001**	−0.029	0.739	0.223	0.086
Psychological abuse^a^	−0.114	0.240	−0.226	0.026	0.048	0.861
Physical abuse^a^	0.034	0.698	**0.223**	**0.016**	0.283	0.263
Sexual abuse^a^	0.130	0.133	0.199	0.027	0.184	0.649
Girls (*N* = 137)						
Controlling behavior^a^	−0.007	0.940	0.086	0.361	−0.134	0.345
Psychological abuse^a^	0.131	0.229	0.055	0.617	0.457	0.143
Physical abuse^a^	0.047	0.645	0.075	0.466	0.031	0.888
Sexual abuse^a^	−0.011	0.940	0.032	0.716	0.098	0.785

a All analyses control for intervention group, youth trauma experiences, food security, and youth age.

Symptoms of parent PTSD was associated with more externalizing behavior (*B* = 0.209, *p* = 0.005) and more days missed from school (*B* = 0.752, *p*  = 0.008) at 8 months for girls. Further, parent symptoms of depression was associated with more experienced stigma (*B* = 0.188, *p* = 0.011) for girls at 8 months. Although not significant at the Bonferroni-adjusted alpha level of 0.017, parent symptoms of depression was associated with more externalizing behavior (*B* = 0.174, *p* = 0.019) for girls at 8 months.

### Relationship of parent IPV experience/perpetration on adolescents experience stigma, behavior, and days missed from school

Parent IPV experience or perpetration at baseline was not related to experienced stigma or missed days from school at 8-month follow-up for boys (Table 4). However, as experience/perpetration of physical IPV increased, externalizing behaviors for boys increased (*B* = 0.223, *p* = 0.016). Parent experience and perpetration of IPV was not associated with adolescent experienced stigma, externalizing behavior or days missed from school for girls at 8-month follow-up.

## Discussion

The analysis provides important insight that contributes to understanding the relationship between parent mental health and IPV and adolescent experienced stigma, externalizing behavior, and school attendance in a post-conflict and humanitarian setting. The findings reinforce the critical role of parents in the health and well-being of their children as they develop through adolescence to adulthood. Specifically, we found that parent's mental health and IPV victimization/perpetration can have a negative impact on several aspects of their children's lives, and the impact is different for boys and girls. For example, in our study the social relationships of adolescents, as reported through experienced stigma (e.g. feeling people act as if they are afraid them, people treat them with less respect than others and called them names or insulted them), were negatively impacted for both boys and girls. Parent's mental health (e.g. depression and PTSD) had a stronger negative effect on girls’ outcomes, including experienced stigma, externalizing behaviors (e.g. fighting, using bad language, being disrespectful), and missed days of school in past 30 days than boys. Parent symptoms of PTSD had a negative effect on boys’ use of externalizing behaviors. Parent's use of physical IPV was significantly related to adolescent boys use of externalizing behaviors at 8-month follow-up.

Our findings are consistent with other studies conducted in conflict and post-conflict settings (Panter-Brick *et al.* 2014). For example, parents in Afghanistan and in our study in eastern DRC prioritized enrolling their children in school (over 90% of adolescents in the parent study were enrolled in school) despite severe economic hardship as revealed through food insecurity in the household. Importantly, our finding demonstrates that young adolescent girls reported missing more school days in the past month at 8-month follow-up compared with boys and the girl's missed school days were associated with the poor mental health of the parents. Our findings are consistent with evidence from Afghanistan that parent mental distress was significantly associated with adolescent reports of symptoms consistent with PTSD and depression (Panter-Brick *et al.* 2014). Adolescents living in post-conflict Uganda and Israel describe a relationship between increased symptoms of PTSD, depression and aggressive behaviors related to war-related trauma exposure and reduced parental attachment (Pat-Horenczyk *et al.* 2007; Okello *et al.* 2015) illustrating the importance of security and support within the home. Our study examined the use of externalizing behaviors, such as fighting, using bad language, and being disrespectful to others by adolescent boys and girls. Our work advances evidence from post-conflict and humanitarian settings, by demonstrating parental symptoms of PTSD and depression negatively impacts both boys and girls – however, girls reported negative impact on multiple outcomes, including experienced stigma, externalizing behavior, and missed school days when compared with boys, were negative outcomes were only in experienced stigma at 8-month follow-up.

Although there is a growing body of evidence that demonstrates the negative and potentially lifelong impact of witnessing IPV and experiencing violence in childhood, evidence remains limited on the impact for young adolescents in post-conflict settings, especially differences by gender. Gender differences in outcomes are important given the strict gender norms that limit girls movement outside the home to participate in activities with supportive peers and other community members. Conflict in families, such as the use of IPV, has been associated with greater difficulties for adults and children in diverse conflict settings, including Afghanistan, where adults and youth reported IPV and the quality of home life as risk and protective factors, respectively, for child mental health (Panter-Brick *et al.* 2014). A study with young adolescents attending school in post-conflict Sri Lanka demonstrated that witnessing IPV and experiencing traumatic events associated with the conflict were significantly related to symptoms of PTSD (Catani *et al.* 2008). In post-conflict Uganda and Afghanistan, research indicates a link between adolescent exposure to war-related traumatic events, IPV and community violence with increased engagement in risk behaviors, such as alcohol/drug use and/or unsafe sex (Catani *et al.* 2009; Klasen *et al.* 2010). Although our analysis found a significant relationship only between physical IPV and boys externalizing behaviors, qualitative interviews previously conducted in these same communities other risks (e.g. alcohol use) to adolescent health, behavior, and social outcomes that were not examined in this secondary analysis (Kohli *et al.* 2015).

Our findings suggest that girls are more vulnerable than boys to the range of negative outcomes (e.g. stigma, poor health, and missed school days) associated with parents’ poor mental health. Study findings indicate boys vulnerability to increased externalizing behavior associated with parent symptoms of PTSD and physical IPV. The age range of adolescents, ages 10–15 years, for the study is important as these ages are consistent with increasing responsibilities of adolescents within and outside the household. Adolescence is an important developmental period when established gender and social norms are being reinforced by parents and other adults. For example, after attending school, adolescent girls return home to take up their responsibilities under the supervision and guidance of the mother or female guardian, including caring for younger children, domestic work such as washing clothes, cleaning the home and gardening – thus requiring significant time daily within the household or housing compound. Additionally, if the mother/female guardian is ill or unable to fulfill her household and caretaking roles, the adolescent girl child is often called upon to take over the domestic responsibilities likely requiring her to be late for or miss the school day. When adolescent boys return from school, they are often responsible for caring for animals or running errands outside the household, thus spending less time in interaction with the parent and more time interacting with peers, neighbors, and other community members. Therefore, gender and social norms are likely important factors in the association between parent mental health and experience/perpetration of IPV and adolescent outcomes, including stigma, externalizing behavior, and school attendance.

### Limitations

This study has important limitations. The study was conducted with young adolescents ages 10–15 years in ten rural villages in one province of eastern DRC, therefore, the findings cannot be generalized to other post-conflict rural villages and urban settings globally. Importantly, several potentially relevant parent and adolescent experiences and behaviors were not included in this analysis but may be important to examine to further understand outcomes. For example, parent alcohol consumption may provide additional insight into factors that influence adolescent health and well-being.

### Implications for future research and programs

Multiple interacting factors that include individual (e.g. childhood exposure to violence/trauma), family (e.g. mothers/fathers mental health, parent–child relationship) and social factors (e.g. social norms about gender equality) influence adolescent health and behavioral outcomes (Werner, 2012). Research evidence has consistently demonstrated that one of the strongest predictor of adult perpetration and/or victimization of IPV is childhood witnessing and experience of IPV (Fulu E *et al.* 2013; Fulu *et al.* 2017). Therefore, primary prevention is an important way forward for addressing mental health and IPV programming in post-conflict and other humanitarian settings. Engaging both parents and young adolescents in examining gender and social norms that maintain and sustain gender inequality has the potential to positively improve health and well-being for adolescents and adults. Primary prevention of violence against women and girls in global humanitarian settings have sought to gain an understanding of social norms and to evaluate social and gender-transformative interventions specifically addressing conceptions of masculinity and femininity (e.g. protecting family honor/dignity, caring for husbands/children, disciplining women if they disobey the husband) as factors that limits girls and boys potential and support families and communities acceptance or tolerance of IPV and other forms of violence against women and girls.

Further, efforts to rebuild and strengthen family and community structures in post-conflict and other humanitarian settings are essential to improved health and well-being for adults and adolescents. For example, RFR is a parent-approved program that engages boys and girls in productive activities outside the home with peers and adult mentors thus providing adolescents with opportunities to learn new skills in animal husbandry, build positive relationship with peers and provide resources from the breeding and selling of the rabbits that can contribute to school fees for self and siblings for example, thus improving their outlook for the future.

Multi-sectoral and multilevel responses are needed to focus on prevention and response programs that engage both mothers and fathers in developing nurturing home environments, positive parenting skills, healthy relationships and communication skills with each other and children. Additionally, engaging with community key stakeholders (e.g. traditional leaders, religious leaders, teachers, health providers, and others in the community, including parents) as leaders to advocate for psychosocial support, including individual and group cognitive processing therapy (CPT) delivered by lay workers that have demonstrated effectiveness with female survivors of sexual violence. Innovations in CPT and other mental health programs are needed to address adult and adolescent symptoms of depression and PTSD, to increase safety and protection services and to address stigma associated with poor mental health and violence.
